# A two-year study of diffused retinal pigment epitheliopathy treated with half-dose photodynamic therapy guided by simultaneous angiography and optical coherence tomography

**DOI:** 10.1038/s41433-018-0284-z

**Published:** 2018-12-07

**Authors:** Yang Liu, Lei Li, Elena Yingqiu Zhu, Yuanzhi Yuan, Wenji Wang, Gezhi Xu

**Affiliations:** 1 Department of Ophthalmology, Eye and ENT Hospital of Fudan University, Shanghai, 200032 China; 2 Shanghai Key Laboratory of Visual Impairment and Restoration, Eye and ENT Hospital, Shanghai Medical College, Fudan University, Shanghai, 200032 China; 3 Melbourne Medical School, University of Melbourne, Parkville, VIC 3010 Australia; 4 Department of Ophthalmology, Zhong Shan Hospital of Fudan University, Shanghai, 200032 China

**Keywords:** Retinal diseases, Eye manifestations, Therapeutics

## Abstract

**Objectives:**

Diffused retinal pigment epitheliopathy (DRPE) is not necessarily the same as chronic central serous chorioretinopathy (CSC), but a severe subgroup under the umbrella of chronic CSC. This study was to evaluate the efficacy and safety of half-dose PDT treating DRPE.

**Methods:**

A retrospective case series design was used. Forty-eight consecutive patients (48 eyes) with DRPE treated with half-dose PDT underwent follow-up at baseline, 3 months, 6 months, 12 months and 24 months. Simultaneous FA, ICGA and OCT were used for the treatment and follow-up. The primary outcomes were the subretinal fluid and best-corrected visual acuity in optical coherence tomography.

**Results:**

Complete fluid absorption was achieved in 95.8% of eyes at 3 months and 100.0% of eyes at 24 months. The baseline logarithm of the minimum angle of resolution (logMAR) BCVA, which was 0.51 ± 0.36, significantly improved to 0.43 ± 0.38 (*p* < 0.001) at 6 months. The boost continued to 0.29 ± 0.37 (*p* < 0.001) at 12 months and 0.19 ± 0.39 (*p* < 0.001) at 24 months. The integrities of the ellipsoid zone (EZ) and interdigitation zone (IZ) improved throughout. Regression analyses showed the BCVA in logMAR was inversely correlated with the EZ (*p* < 0.01) and IZ (*p* < 0.01). The recurrence rate was 6.3%. No severe complications were witnessed.

**Conclusions:**

In 48 eyes with DRPE, simultaneous angiography and OCT facilitated a more comprehensive guidance for half-dose PDT treatment and follow-up. The BCVA improvement occurred at 6 months, which may be attributed to the restoration of the outer retinal structure.

## Introduction

**Fig. 1:**
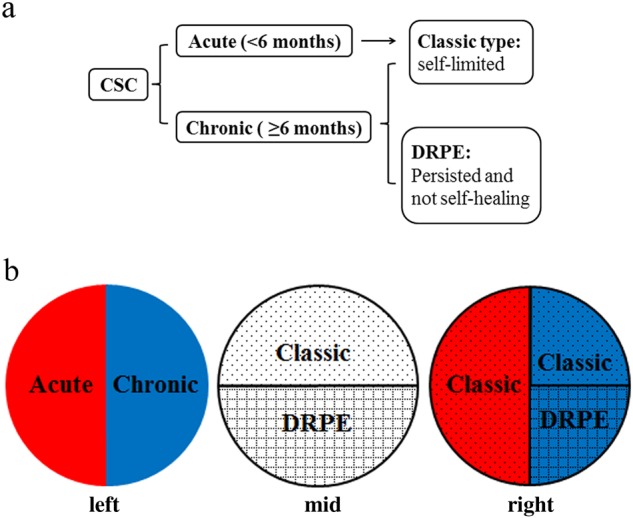
CSC can be classified to acute or chronic type, based on symptom duration, and can also be subgrouped into classic and DRPE, based on the situation of RPE. In fact, acute type was not always corresponded to classic, as well as the chronic to DPRE. Just as the tree diagram showed, there exists some cases presented with self-limited tendency but recurred lasting for more than 6 months. The widespread RPE decompensation was not witnessed in such recurrent type. The more than 6 months duration was only a necessary but not sufficient condition, while the diffused RPE abnormality was the gold standard to confirm the diagnosis of DRPE. The most common classification of CSC is acute (**left**, red zone) or chronic type (**left**, blue zone), 6 months as a dividing point. The situations of RPE can also subgroup CSC into classic (**mid**, dot zone) and DRPE (**mid**, grid zone). All acute cases (**right**, red dot zone) can be categorised to classic type. Recurred cases (**right**, blue dot zone) with classic character last for more than 6 months, supposed to be classified to chronic CSC. DRPE type (**right**, blue grid zone) lasts for more than 6 months and presents with widespread RPE decompensation. The pie has no meaning in proportion, but a schematic diagram

Central serous chorioretinopathy (CSC) is defined as a serous exudative detachment of the retina, usually confined to the macula, and it exhibits a variety of visual manifestations [1]. If CSC lasts more than 3–6 months, it is considered chronic. CSC can also be classified into the classic type and diffused retinal epitheliopathy (DRPE), based on the status of the retinal pigment epithelium (RPE) [2]. The classical type is accompanied with a largely favourable natural history, characterised by a relatively well-preserved retina [2, 3]. DRPE involves extensive RPE disruptions and diffused leakage, leading to a poor prognosis due to anatomical damage. As there are eyes with CSC lasting for more than 6 months but manifesting with recurrent classic characteristics, chronic CSC is not necessarily the same as DRPE. For this reason, we applied this classification to chronic CSC in this study and evaluated only patients presenting with the manifestations of DRPE. DRPE represented the specific subtype of chronic CSC in this study, where it largely differed from the classic type due to severe widespread RPE decompensation and a poor prognosis (Fig. 1).

Years ago, photodynamic therapy (PDT) with verteporfin was proposed for treating CSC [4]. In terms of DRPE treatment, a variety of medical treatment modalities have been proposed, including drugs, thermal laser and PDT. However, only PDT has been able to solve the underlying choroidal hyperpermeability problem [5]. The mechanism of action involves halting choriocapillary hyperperfusion in the short term and remodelling the choroidal vascularisation in the long term [6]. However, standard PDT may have irreversible complications when used for treating CSC and DRPE [7, 8]. Modified PDT regimens were found to be associated with improved treatment outcomes and a lower incidence of adverse effects [9–14]. In comparing different regimes, PDT with half-dose verteporfin appeared to be the optimal choice.

Since DRPE presents with long-standing, severe, widespread RPE decompensation, its treatment remains challenging, and the outcomes should be studied [15]. In this research, we aim to evaluate the 24-month efficacy and safety of half-dose PDT for DRPE, guided by simultaneous optical coherence tomography (OCT), fluorescein angiography (FA) and indocyanine green angiography (ICGA).

## Methods

This retrospective study was performed at the Department of Ophthalmology, Eye & ENT Hospital of Fudan University, Shanghai, China. Patients presenting with chronic DRPE from April 2011 to December 2015 were recruited and followed up for at least 24 months after half-dose PDT administration. All treatments were performed by a retinal specialist (LL).

The patient inclusion criteria were as follows:

1. Visual impairment, central scotoma, metamorphopsia, micropsia, dyschromatopsia or hypermetropisation for ≥6 months;
2. Subretinal fluid (SRF) involving the fovea, as confirmed by OCT, with multiple, widespread or subtle leaks detected by FA, and abnormal choroidal vascular dilation or hyperpermeability detected by ICGA.

The exclusion criteria were as follows:

1. Choroidal neovascularisation (CNV), polyploidal choroidal vasculopathy or other maculopathy;
2. Prior use of corticosteroids in any form;
3. Thermal laser photocoagulation in the last 6 months or history of intravitreal injection of anti-vascular endothelial growth factor agents;
4. Any contraindications for angiographic or verteporfin dyes.

Pre-PDT ocular examinations included measurement of the best-corrected visual acuity (BCVA), using Early Treatment Diabetic Retinopathy Study charts; the outcomes were presented in logarithm of the minimum angle of resolution (logMAR) scale. Dilated fundus examinations included fundoscopy and slit-lamp biomicroscopy with a 120-D superfield lens. Simultaneous FA, ICGA and OCT were performed using the Spectralis^®^ HRA + OCT system (Heidelberg Engineering, Heidelberg, Germany).

To obtain the OCT images, horizontal and vertical linear and volume scans of the fovea and abnormal areas showed on angiography were performed. The external limiting membrane (ELM), ellipsoid zone (EZ) and interdigitation zone (IZ) were morphologically classified as absent, fragmented or continuous using the grading scheme proposed by Fujita et al. [16]. The central neural retinal thickness (CNT) was assessed by manually measuring the distance between the internal limiting membrane and photoreceptor outer segments in the central fovea [17, 18].

On FA, diffuse leakage was defined as more than three foci or a widespread active leak, and subtle leakage as non-demonstrable active foci. ICGA was used to assess the two following parameters: choroidal vascular hyperpermeability pre- and post PDT [19] and choriocapillary perfusion post PDT [20]. Each classification was evaluated by YL and LL, who were masked to the patients’ characteristics and treatment.

After obtaining written informed consent, 3 mg/m^2^ of verteporfin was infused over 8 min, followed by laser delivery at 10 min after the starting infusion. The 689-nm laser (Opal Photoactivator; Lumenis, Beijing, China) was applied for 83 s at an intensity of 50 J/cm^2^, as previously described by Chan et al. [8]. The laser-targeted leakages on FA and choroidal hyperpermeability on ICGA, as well as sites with fluid identified by the Spectralis HRA + OCT system, even without positive manifestations on the angiographs. The spots were intended to cover the greatest linear dimension of the abnormal area while avoiding any overlap on the fovea. An ocular fundus laser lens (Ocular Inc., Bellevue, WA, USA) was used to achieve spot diameters of 1100–5400 μm. The patients were instructed to avoid light exposure for 5 days post PDT.

All the patients were instructed to attend for follow-up at 0, 3, 6, 12 and 24 months post PDT. BCVA and OCT were analysed at all visits. FA and ICGA were examined at 3 months post PDT. Additional FA and ICGA were carried out if active lesions were persistent or recurrent during the follow-up period.

SPSS software version 20.0 (IBM Inc., Armonk, IL, USA) and GraphPad Prism 6.0 (GraphPad Software Inc., La Jolla, CA, USA) were used for all data analyses and picture constructions. The paired *t*-test, analysis of variance and non-parametric Wilcoxon signed-rank test were used to compare differences. Linear regression analyses were performed to assess the associations between the logMAR BCVA and EZ and IZ. A *p*-value < 0.05 was considered to indicate statistical significance.

## Results

### Patient demographics

Forty-eight eyes in 48 Chinese patients, 40 males (83.3%) and 8 females (16.6%), were included in this study. The mean ± standard deviation age of the patients was 47.8 ± 7.1 years (range: 38–66 years). The median duration of symptoms was 21 months (range: 6–120 months). Fourteen eyes (29.2%) had experienced more than one episode of CSC prior to PDT. The duration of follow-up was 24 months after the initial PDT.

### PDT parameters and number of treatment sessions

The average PDT spot diameter was 3220 μm (range: 1100–5400 μm). The median number of spots per eye was 2 (range: 1–4). The numbers of spots were 1 in 19 eyes (39.6%), 2 in 18 eyes (37.5%) and ≥3 in 11 eyes (22.9%). The median number of PDT sessions was one (range: 1–2); 44 eyes (91.7%) achieved complete resolution of fluid in one treatment session. Four eyes (8.3%) underwent two treatment sessions, among which, 3 eyes (6.3%) exhibited recurring fluid. All 14 eyes (29.2%) with a history of symptom recurrence prior to PDT achieved complete resolution of fluids after a single treatment session, and this lasted for the entire follow-up period.

### Angiographic findings

The baseline FA revealed diffuse fluorescein leakage in 36 eyes (75.0%) and subtle or oozing leakage in 12 eyes (25.0%). Middle-phase ICGA revealed intermediate hyperfluorescence in 9 eyes (18.7%) and intense hyperfluorescence in 39 eyes (81.3%). At 3 months post PDT, there were no signs of leakage in 42 eyes (87.5%), subtle leakage in 5 eyes (10.4%; no serous fluid on OCT) and persistent pigmental epithelial detachment (PED) pooling was observed in 1 eye (2.1%). The middle-phase ICGA revealed no hyperfluorescence in 35 eyes (72.9%), intermediate hyperfluorescence in 6 eyes (12.5%) and intense hyperfluorescence in 7 eyes (14.6%).

### OCT findings

**Fig. 2:**
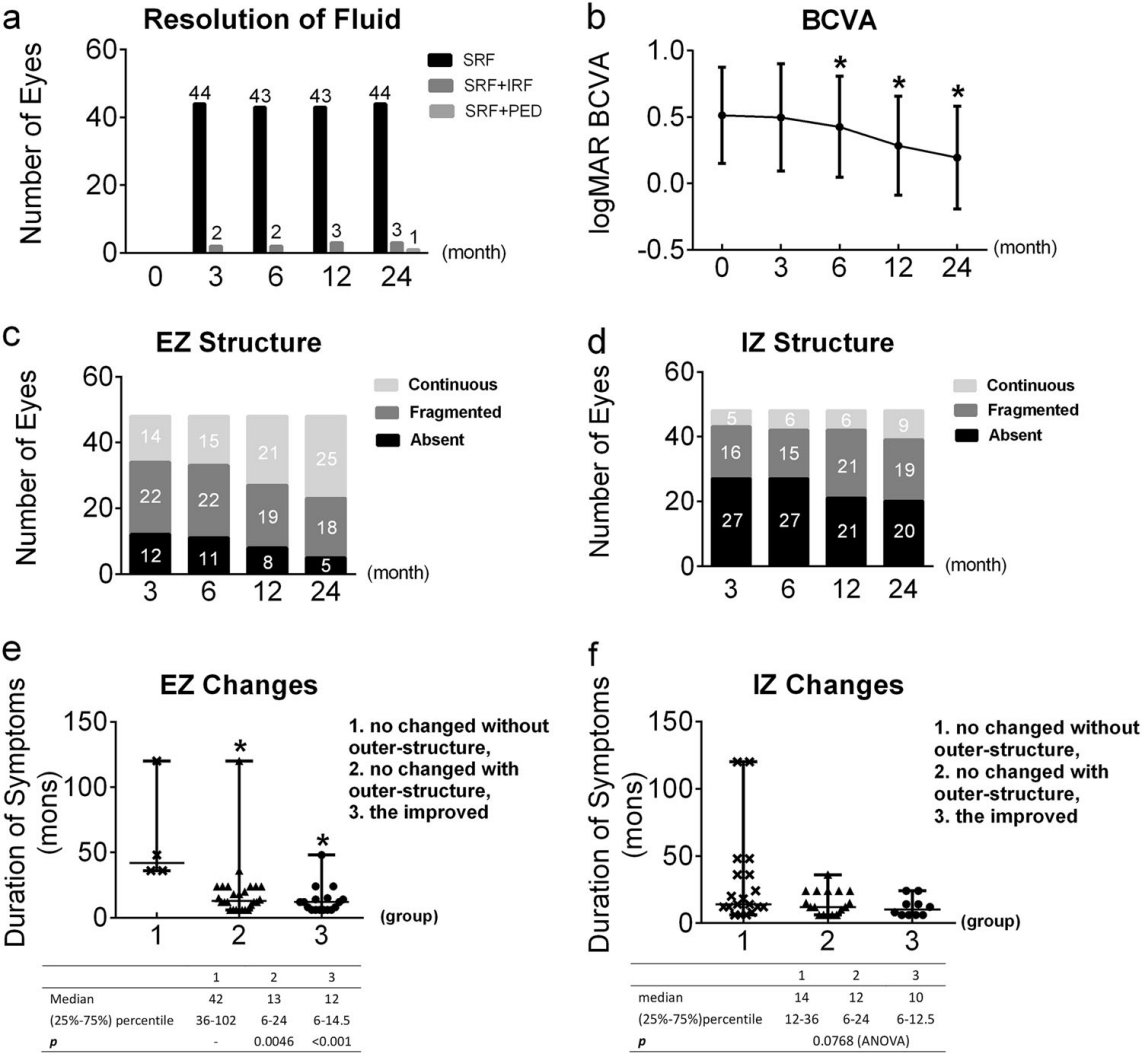
The changes in subretinal fluid and best-corrected visual acuity (BCVA) during whole follow-up (**a**, **b**) resolution of fluid and average BCVA throughout. Bars are the standard error of the mean. The BCVA is significantly better at 6, 12 and 24 months than at baseline. **c**, **d** Numbers of integrity of ellipsoid zones (EZ) and interdigitation zones (IZ) on optical coherence tomographic at 3, 6, 12 and 24 months. The numbers of eyes with fragmented and continuous EZ and IZ gradually increased after treatment. In EZ, eyes in group 1 had longer duration than the rest two groups. The rest has no difference between each other. And no statistical difference was found in different groups of IZ (**e**, **f**). *Compared with baseline values *p* < 0.05

At baseline, all eyes (100%) had SRF involving the central fovea, 3 eyes (6.25%) also had intra-retinal fluid (IRF) and 1 eye (2.1%) also had extensive PED (affecting an area larger than one entire diameter of the papillary disc). Overall, complete fluid absorption was achieved in 46 eyes (95.8%) at 3 months, 45 eyes (93.8%) at 6 months, 46 eyes (95.8%) at 12 months and 48 eyes (100.0%) at 24 months (Fig. 2). Two eyes with isolated SRF were retreated at 6 and 12 months, respectively, due to recurrence of SRF; in both cases, the fluid absorbed at 1 month post PDT. One eye with recurrent SRF and IRF was retreated at 6 months, and the fluid resolved 3 months later. The eye that presented with SRF and PED at baseline had complete resolution of the SRF after the initial treatment.

Forty-three eyes (89.6%) presented with continuous ELM throughout. Five eyes (10.4%) presented with fragmented ELM at baseline. Four eyes’ ELMs remained fragmented after treatment until 24 months, while the other presented with no changes at 3 months, but it was continuous from 12 months until the final visit. The manifestations of EZ and IZ from 3 to 24 months are shown in Fig. 2. The numbers of eyes in which the EZ or IZ was fragmented or continuous increased gradually during the follow-up period; compared with the 3 months’ manifestation, the EZ changed slightly at 6 months (*p* = 0.414); significant structural improvements started at 12 months (*p* = 0.008) and proceeded to 24 months (*p* < 0.001). The IZ changed slightly at 6 and 12 months (*p* = 0.705 and 0.052), and clear recovery emerged up to 24 months (*p* = 0.012). The relation of outer retinal structure changes and durations of symptom were assessed, and found eyes with shorter duration of DRPE reserved the reversibility of EZ (Fig. 2). The CNT remained stable throughout the whole follow-up; it was 116.4 ± 33.0 μm at baseline, 112.6 ± 32.7 μm at 3 months, 114.0 ± 31.9 μm at 6 months, 115.3 ± 32.1 μm at 12 months and 118.9 ± 32.9 μm at 24 months (*p* > 0.05).

### Changes in visual function

The baseline BCVA, in logMAR, was 0.51 ± 0.36 (range: 0.00 to 1.40). The BCVA was unchanged at 3 months, with a mean logMAR of 0.50 ± 0.40 (range: 0.00 to 1.40; *p* = 0.336). It started to improve to 0.43 ± 0.38 (range: –0.18 to 1.40; *p* < 0.001) at 6 months. The boost continued to 0.29 ± 0.37 (range: −0.2 to 1.10; *p* *<* 0.001) at 12 months and 0.19 ± 0.39 (range: −0.3 to 1.00; *p* < 0.001) at 24 months (Fig. 2). The average improvement in BCVA at the final visit was 3.2 lines. The visual acuity improved by ≥2 lines in 41 eyes (85.4%) 24 months after treatment, ≥4 lines in 17 eyes (35.4%) and no more than 1 line in 7 eyes (14.6%). None of the eyes experienced a worsening in visual acuity.

**Fig. 3:**
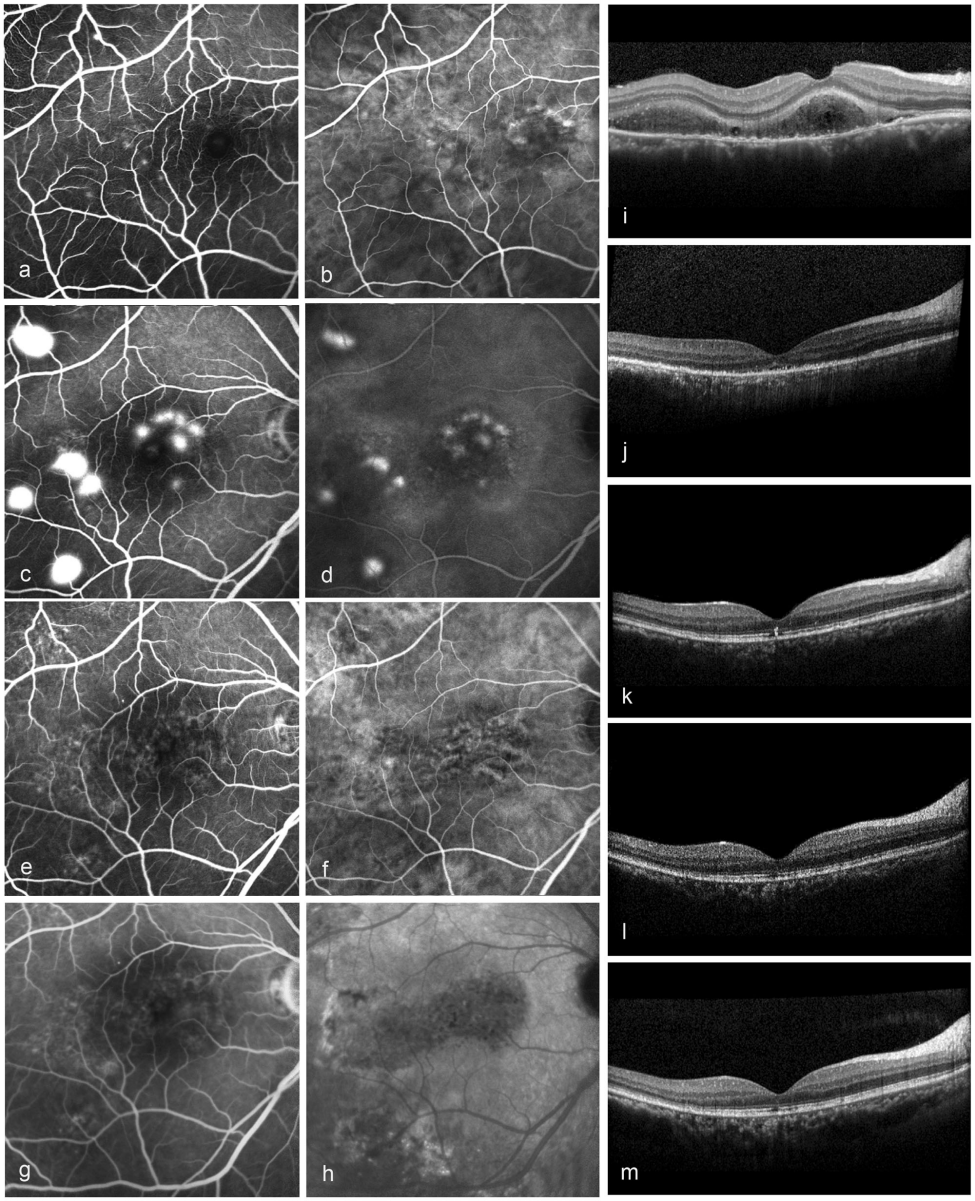
A 38-year-old male with diffused retinal pigment epitheliopathy for 14 months and deteriorated over recent 1 month. **a**, **b** Early-phase angiography showed diffused hyperfluorescence across the posterior region in fluoroscein angiography (FA) and choroidal vascular dilation in indocyanine green angiography (ICGA). **c**, **d** Mid-phase images showed at least 11 active fluorescein leaks and intense choroidal hyperpermeability staining. **e**–**h** At 3 months, the FA and ICGA images showed no active fluorescein leaks despite the persistent choroidal vascular dilation. Optical coherence tomograpgy images: **i** at baseline, external limiting membrane (ELM): fragmented; **j** at 3 months, ELM: fragmented, ellipsoid zone (EZ): absent, interdigitation zone (IZ): absent; **k** at 6 months, ELM: fragmented, EZ: fragmented, IZ: absent; **l** at 12 months, ELM: continuous, EZ: fragmented, IZ: absent; **m** at 24 months, ELM: continuous, EZ: fragmented, IZ: fragmented. The subretinal fluid absorbed at 3 months without recurrence, and best-corrected visual acuity (logarithms of the minimum angle of resolution) was 0.15, 0.40, 0.22, −0.08 and −0.20 at each visit

**Fig. 4:**
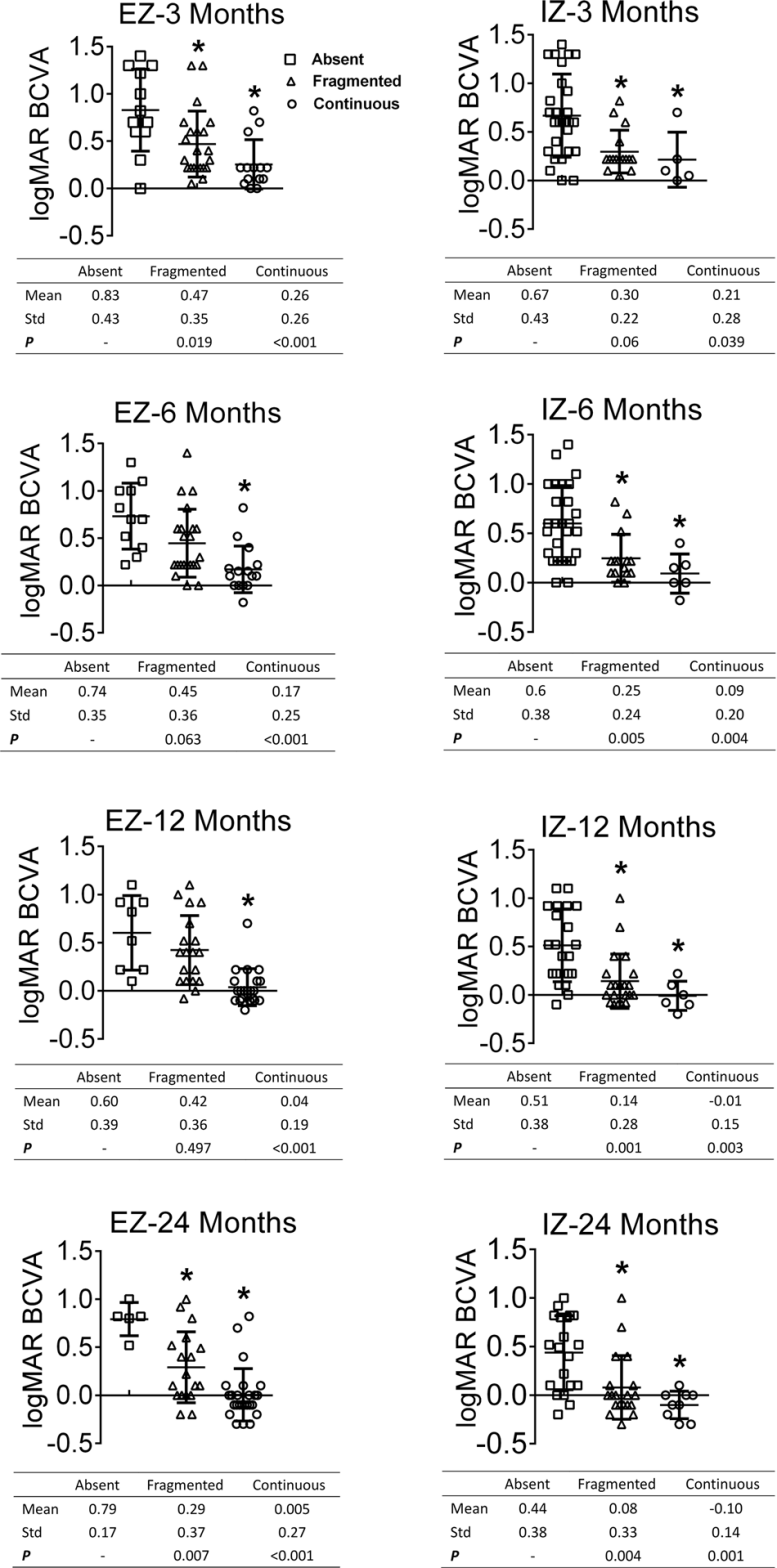
Scattergram showing the best-corrected visual acuity (BCVA) in logMAR as a function of photoreceptor manifestation on optical coherence tomography after half-dose photodynamic therapy. Note that error bar means variations of BCVA. Different symbols (squares, triangles and circles) represent different status of ellipsoid zones (EZ) and interdigitation zones (IZ). Eyes with more intact integrity of EZ and IZ possessed with better BCVA. *Compared with absent EZ or IZ, *p* < 0.05

During the follow-up, 43 eyes (89.6%) with continuous ELM had a mean BCVA enhancement for 3.3 lines, while 4 eyes (8.3%) with fragmented ELM had an enhancement for 1 line. One eye with fragmented ELM recovered to become continuous, along with obvious improvement for 4 lines (Fig. 3). Similarly, the eyes with a more intact integrity of EZ and IZ possessed better BCVA (Fig. 4). The BCVA in logMAR was inversely correlated with the EZ and IZ at the 3-, 6-, 12- and 24-month visits (*p* < 0.01).

### Safety

None of the patients developed any systemic adverse events due to the verteporfin infusion. At 3 months post PDT, ICGA revealed mild nonperfusion (grade 1) in 9 (18.8%) eyes and normal choriocapillary perfusion (grade 0) in 39 (81.2%) eyes. The CNT remained stable during the follow-up period. There were no cases of further neurosensory retinal atrophy or secondary CNV during the follow-up period.

## Discussion

Based on the RPE status, CSC was classified as classic type and DRPE. Classic CSC may occur only once or more than once, but it regresses spontaneously [2, 3]. When recurrent symptoms last for more than 6 months, the classic type is regarded as chronic CSC. DRPE, as a more severe type of this disorder, has no inclination for self-healing and always lasts for more than 6 months. Consequently, DRPE belongs to the category of chronic CSC, but chronic CSC is not always identical to DRPE. To specifically exclude the classic type (≥6 months) of chronic CSC, we recruited the severest type of chronic CSC.

Since 2003, many studies have evaluated the long-term efficacy of PDT for chronic CSC [5, 17, 21–25]; however, only a few studies have discussed the efficacy and safety of PDT in treating DRPE. We previously reported the favourable outcomes of half-dose PDT for chronic CSC, including the classic type and DRPE [26]. In this study, patients with widespread RPE decompensations were recruited and only 48 eyes met the criterion.

Given the complexity of DRPE, PDT radiation was expected to cover all the active spots to guarantee the efficacy of the treatment. In chronic CSC, active lesions are illustrated from leakage on FA, choroidal hyperfluorescence on ICGA. When it comes to DRPE, except for the classic former two, it should include the sites with fluid demonstrated by the OCT, though uncertainty leaking shown in correspondence areas with angiography [27]. For the fluid comes from active lesion, the PDT laser spot should cover the area. This kind of non-classic active lesions is easy to be missed by a therapist during treatment. Fortunately, the simultaneous FA, ICGA and OCT system allows the interesting stratus on OCT and leaking sites on angiographs, corresponding to each other, to be visualised [28]. In other words, when the active angiographic manifestation is not clear, a therapist can scan the simultaneous OCT through suspicious leaking sites to determine whether there is fluid. This helps to unveil all active lesions, to prevent them from being missed by a therapist during treatment; as a result, it promotes the resolution of fluid post PDT. In our study, complete fluid absorption was achieved in 95.8% of eyes at 3 months and 100.0% of eyes at 24 months. The recurrence rate was 6.3%.

After the resolution of fluid, the recovery of the microstructure on OCT and BCVA represented major indicators reflecting the efficacy of half-dose PDT. The ELM, EZ and IZ, the common indicators of the microstructure [15, 16, 29], were also used to assess the microstructural recovery (Fig. 2). As the changes in the ELM were slight throughout the follow-up, only the improvement of BCVA on different ELM statuses was compared. Gutiérrez-Hernández et al. [29] previously suggested that an intact ELM could forecast the possibility of visual recovery. We also found that eyes with continuous ELM gained greater improvements. One eye with a fragmented ELM at baseline recovered with an apparent BCVA benefit (Fig. 3). Although we did not find that every injured ELM could still regenerate post half-dose PDT, this specific reappearance of the ELM may predict the likelihood of BCVA recovery.

**Fig. 5:**
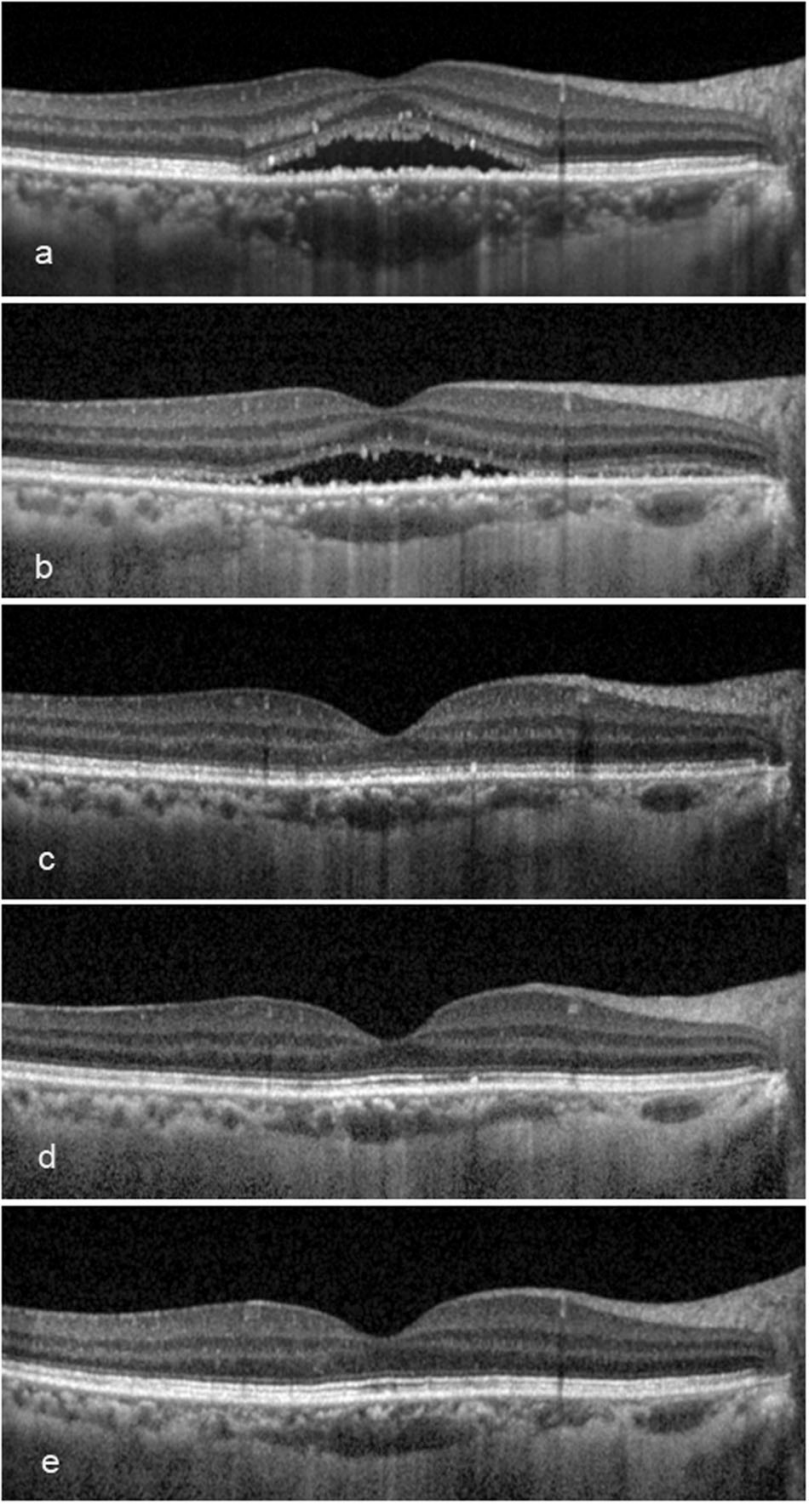
The outer retinal microstructure recovery post treatment of a 43-year-old female with symptom duration for 6 months. **a** At baseline, ellipsoid zone (EZ): absent, interdigitation zone (IZ): absent; **b**, **c** at 3 and 6 months, EZ: fragmented, IZ: absent; **d** at 12 months, EZ: continuous, IZ: absent; **e** at 24 months, EZ: continuous, IZ: fragmented. The subretinal fluid at baseline had been partly absorbed at 3 months, and completely absorbed at 6 months. The external limiting membrane presented continuous along the whole follow-up. The best-corrected visual acuity (logarithms of the minimum angle of resolution) was 0.22, 0.10, 0.00, −0.10 and −0.30 at each visit

The baseline morphologies of EZ and IZ were not evaluated because of the presence of fluid. By comparing the morphologies at each visit, it was evident that the integrities of the EZ and IZ improved. Only in EZ, shorter duration appeared to reserve the ability to reversibility (Fig. 2). A longer follow-up and larger sample size study may be needed to confirm the non-significance in IZ changes. The BCVA in logMAR was better in eyes with more intact EZ and IZ structures (Fig. 4). Based on the regression analyses, the improvements in function seem to have been due to the renewed structural integrities (Fig. 5).

The average BCVA remained unchanged from baseline to 3 months, but it started to improve significantly at 6 months. The occurrence of BCVA improving appeared later than in other studies [16, 21–25], which implied that the recruited patients manifested more severe baseline conditions than are apparent in the general chronic CSC, and thus, they required a longer period to recover. The improvement continued until 24 months, confirming the efficacy of simultaneous, ICGA and OCT-guided PDT.

Secondary CNV and neural retinal atrophy are the severest side effects of full-dose PDT. Previous research has established that choroidal hypoperfusion increases the risk of developing CNV [5, 6, 8, 30, 31]. Our results demonstrated that, at 3 months, only a mild nonperfusion effect (grade I) was observed in 16.7% of eyes, and none of the patients had developed CNV by the 24-month follow-up visit. The CNT was evaluated to reflect the condition of the neural retina. We applied Spectralis^®^ HRA + OCT, which can automatically scan the same retinal stratus at different visits, and found that the CNT remained stable until 24 months.

The limitations of this study included the small sample size, retrospective design and lack of control group. In future investigation, other examinations, including OCT angiography, multifocal electroretinography and microperimetry could provide further valuable information on the efficacy of the treatment. Researchers could also consider assessing the morphological and functional states of the choroid.

## Conclusion

Simultaneous FA, ICGA and OCT facilitated more comprehensive guidance for half-dose PDT treatment in all 48 eyes with DRPE, ensuring its efficacy and safety meantime. The BCVA improvement, which emerged at 6 months, later than general chronic CSC, appeared to be attributable to the restoration of the outer retinal structure.

### Summary

#### What was known before

- Since 2003, many studies have evaluated the long-term favourable efficacy of PDT for chronic CSC.

#### What this study adds

- However, only a few studies have discussed the efficacy and safety of PDT in treating DRPE. In this study, only patients with widespread RPE decompensation were recruited. In eyes with DRPE, simultaneous FA, ICGA and OCT facilitated more comprehensive guidance for half-dose PDT treatment, ensuring its efficacy and safety. Although the visual acuity improved at 6 months, latter than other studies, it did happen, along with the recovery of retinal structure.
